# Hand hygiene and aseptic techniques during routine anesthetic care - observations in the operating room

**DOI:** 10.1186/s13756-015-0042-y

**Published:** 2015-02-06

**Authors:** Veronika Megeus, Kerstin Nilsson, Jon Karlsson, Bengt I Eriksson, Annette Erichsen Andersson

**Affiliations:** The Sahlgrenska Academy, Institute of Health and Care Sciences, University of Gothenburg, Box 457, 405 30 Gothenburg, Sweden; Department of Anesthesia, Surgery and Intensive Care, Sahlgrenska University Hospital/Östra, Diagnosvägen 11, 416 85 Gothenburg, Sweden; Department of Orthopedics, Sahlgrenska Academy, Sahlgrenska University Hospital, 413 45 Gothenburg, Sweden; The Sahlgrenska Academy, Institute of Clinical Sciences, University of Gothenburg, Gothenburg, Sweden; The Swedish Institute for Health Science, Lund, Sweden

## Abstract

**Background:**

More knowledge is needed about task intensity in relation to hand hygiene in the operating room during anesthetic care in order to choose effective improvement strategies. The aim of this study was to explore the indications and occurrence of hand hygiene opportunities and the adherence to hand hygiene guidelines during routine anesthetic care in the operating room.

**Methods:**

Structured observational data on hand hygiene during anesthetic care during 94 surgical procedures was collected using the World Health Organization’s observational tool in a surgical department consisting of 16 operating rooms serving different surgical specialties such as orthopedic, gynecological, urological and general surgery.

**Results:**

A total of 2,393 opportunities for hand hygiene was recorded. The number of hand hygiene opportunities when measured during full-length surgeries was mean = 10.9/hour, SD 6.1 with an overall adherence of 8.1%. The corresponding numbers for the induction phase were, mean =77.5/h, SD 27.4 with an associated 3.1% adherence to hand hygiene guidelines. Lowest adherence was observed during the induction phase before an aseptic task (2.2%) and highest during full-length surgeries after body fluid exposure (15.9%).

**Conclusions:**

There is compelling evidence for low adherence to hand hygiene guidelines in the operating room and thus an urgent need for effective improvement strategies. The conclusion of this study is that any such strategy should include education and practical training in terms of how to carry out hand hygiene and aseptic techniques and how to use gloves correctly. Moreover it appears to be essential to optimize the work processes in order to reduce the number of avoidable hand hygiene opportunities thereby enhancing the possibilities for adequate use of HH during anesthetic care.

## Background

Hospital-acquired infections (HAI) are one of the greatest challenges of modern healthcare. They cause unnecessary patient suffering [1,2] and increase the risks for morbidity and mortality [3,4]. In addition, HAI lead to prolonged hospital stays and increased costs [5] at a time when most healthcare systems are struggling with limited resources. HAI contribute to the increased use, overuse and misuse of antibiotics and thereby promote the development of antimicrobial resistance (AMR) [6,7]. The rapid development of AMR poses a global threat to human health as antimicrobials for treating HAI have become an endangered resource [8]. Hand hygiene (HH) has been recognized as one of the most important and cost-effective measures to prevent HAI, and the introduction of the alcohol-based hand rub has facilitated the adoption of HH practice at the point of care [9-11]. A systematic review of HH in intensive care units (ICU) and general wards showed a mean compliance rate of 40%, and the lowest compliance rates were found in ICUs and among physicians [12]. Factors reported by healthcare professionals that are associated with non-adherence in general wards are: high workload, insufficient time, inaccessibility of HH products, skin irritation, HH not being a prioritized task, forgetfulness, lack of scientific information and skepticism concerning the importance of HH [13,14]. The World Health Organization (WHO) has produced evidence-based guidelines on HH in healthcare [15]. A standardized observational method [16] has been developed along with a conceptual framework “My five moments for hand hygiene” that links and explains scientific evidence to HH recommendations in a user-friendly way. These guidelines have been developed as tool to understand, train, monitor and report HH [17] and can be used as part of implementation strategies aimed at improving HH practice.

When initiating data collection for the present study in 2012, only very few clinical studies had been published in terms of the implementation of HH in the operating room (OR) setting [18-20]. The data showed low adherence, 2 -18% to HH guidelines and relatively high numbers of opportunities for HH 35-41/h during anesthetic care were reported [18]. Moreover, a series of studies by Loftus et al. identified the hands of anesthesia providers as vectors of cross-transmission between equipment within the anesthesia work area and the patients’ medical devices [3,21,22]. During the last years there has been an increased interest in studying and reporting HH practice in the OR and in particular during anesthetic care [23,24]. Other studies have focused more on environmental contaminations in the OR and cleaning practices [25] and have investigated the frequency of hand to surface contacts in the anesthesia work area. These studies found high numbers of environmental interactions in combination with few so-called HH events [26,27].

In order to select effective improvement strategies, more knowledge is needed about task intensity in relation to HH practice and the relationship to HH indications and opportunities as they occur in the OR during routine anesthetic care. Thus, the aim of this study was to explore and describe the indications and occurrence of hand hygiene opportunities (HHOs) and the adherence to Swedish national HH guidelines [28] during routine anesthetic care in the OR.

## Methods

### Setting

The study was set in a general hospital in west Sweden with 460 in-patient beds and performing approximately 9,970 surgeries/year. The surgical department consisted of 16 ORs and served different surgical specialties including orthopedic, gynecological, urological and general surgery. The ORs are of varying sizes but interiorly of similar design. Each OR has two dispensers of alcohol-based hand rub. One of them is situated on the side of the drug trolley close by the anesthesia machine and the other is attached to the wall near a computer used by the OR nurses and nursing assistants. Disposable gloves are available close to the workstation. In the preoperative center, dispensers of alcohol-based hand rub are situated on the wall at every bedside. The department has written HH guidelines available on the hospital’s intranet. These mandatory instructions are in line with the Swedish national guidelines produced by the Swedish National Board of Health and Welfare’s [28] regulations on basic hygiene, (see list below) as well as the World Health Organization Guidelines on Hand Hygiene in Health Care [15].

### The Swedish National Board of Health and Welfare’s regulations on basic hygiene in healthcare

*“In conjunction with examinations, care and treatment or any other direct contact with patients, healthcare and medical staff must observe the following in order to limit the risk of care-related infections:*

- *Working clothes must have short sleeves.*
- *Working clothes must be changed every day and more frequently if necessary.*
- *Hands and forearms must be free from watches and jewelry.*
- *Hands must be disinfected with an alcohol-based hand rub, or some other agent with the corresponding effect, immediately before and after every direct contact with a patient.*
- *Hands must be disinfected both before and after using gloves.*
- *If they are visibly dirty, hands must be washed with water and liquid soap before being disinfected.*
- *When caring for a patient with gastroenteritis, hands must always be washed with water and liquid soap before being disinfected.*
- *Hands that have been washed must always be dry before being disinfected.*
- *A disposable apron made of plastic or a protective coat must be used if there is a risk that working clothes will come into contact with bodily fluids or any other biological material.*
- *Protective disposable gloves must be used in the event of contact with or the risk of contact with bodily fluids or any other biological material.*
- *Protective gloves must be removed directly after a working procedure and replaced between different working procedures* [28]*”.* Author translation.

At the study site, the adherence to HH is monitored on a regular basis every month using self-reported data in combination with direct observations executed by the ward manager as apart of the hospital’s quality improvement program.

### Sample

Staff working on the ward included nurse anesthetists, instrument nurses, nursing assistants, anesthesiologists, surgeons and students. All members of these categories were observed if actively taking part in anesthetic care procedure.

### Data collection

Opportunities and indications for and practice of HH during anesthetic care were monitored using a modified version of the WHO’s HH observational method [16]. The modification consisted of the additional recording of the type of indication for HH and detailed information on glove use. The protocol has been tested and used in a previous study set in the OR [20]. An HH action was defined as the use of an alcohol-based hand rub in relation to an HHO. The amount of product used and the duration of application were not recorded. An HH indication is defined as the reason for why the hand hygiene action is required at a specific point of care and is synonymous with ” a moment” as in “My five moments for hand hygiene” [17] see Table 1. An HH opportunity is the time span between two risk-prone hand-surface contacts when one or more of the five indications/moments 1–5 apply [16].

**Table 1:** **A short description of the content of “My five moments for hand hygiene” and one example given within brackets**

**Moment**	**Descriptions**
***1***	***Before patient contact*** *(touching the door handle and then shaking the patient’s hand). Typically this moment for HH occurs between the last hand-surface contact with an object belonging to the so-called healthcare zone and the first within the patient zone.*
***2***	***Before an aseptic task*** *(manipulating or inserting a venous access line). This moment might occur after touching the patient’s intact skin. HH at this moment aims at preventing colonization and HAI. For some aseptic procedures glove use is indicated, and in those cases HH is required before donning the gloves.*
***3***	***After body fluid exposure risk*** *(inserting an intravenous catheter and then preparing a syringe). Protective gloves must be used when there is a risk for contamination of hands with body fluids. Gloves must be removed and HH carried out before proceeding with, after, and/or between different work procedures.*
***4***	***After patient contact*** *(after shaking the patient’s hand and then touching the computer keyboard). This HH action will reduce the risk of dissemination to the healthcare environment and occurs when moving from the patient zone to the healthcare zone.*
***5***	***After contact with patient surroundings*** *(after touching the patient’s bed linen and then touching the door handle) This moment is a variant of moment 4 and HH is implemented for the same reasons.*

One trained observer (VM), a registered nurse specialized in perioperative nursing carried out all observations. The observer had no prior connection to the study site. The observer was trained by one of the senior authors (AEA). The training sessions included studies of the WHO manual and reviews of the evidence base for HH.

The training sessions were set in another hospital and were not included in the study in order to allow free discussions between the trainer and the trainee. During the training session inter-rater concordance was assessed.

Using a single observer meant that it was necessary to select the items that were going to be observed, as one observer cannot manage comprehensive observations including all the HHOs that occur in the OR. The decision was taken to prioritize observations of opportunities for HH in relation to aseptic tasks during anesthetic care as these procedures are considered to be risk-prone [29]. In addition, observations of the risk of hand transmission of microorganisms were recorded. For example, if after manipulation of the airway, no HH was carried out and the health professional subsequently touched a clean site such as stopcocks, this was recorded as a risk for transmission of microorganisms as well as missed HHOs.

To avoid selection bias, the ORs to be observed were randomly selected (“picking from a hat”) each morning. The observations were initiated when the anesthesia providers started to prepare for the coming procedures. When the patients arrived at the study site they were prepared at a preoperative center before being admitted to the OR. On some occasions the patients received intravenous lines as well as regional anesthesia in the preoperative center, hence three observational sessions took place at the preoperative center. The OR staff were aware of being observed for a patient safety study, without knowing however which specific items were of interest.

Initial data assessments of the 43 full-length operations showed that task intensity was highest during the induction phase in contrast to the maintenance phase. For this reason, the following 51 observational sessions were dedicated to the induction phase. The original observational protocol included only invasive aseptic procedures. The following 51 observational sessions also included recordings of HHOs in relation to activities 1, 4 and 5 according to WHO “My five moments for hand hygiene”. The observations of the induction phase were limited to the period between the patient’s arrival in the OR to the anesthesia-ready time after completed induction. The maintenance phase was defined as the period starting from anesthesia-ready time to the end of surgery/wound closure.

### Data and statistical analysis

Adherence to guidelines was calculated by dividing the number of HH actions by the total number of opportunities. An opportunity was defined as a situation requiring hand disinfection. Adherence and the occurrence of HHOs were stratified by professional category and indication. The HHOs were analyzed and categorized according to “My five moments for hand hygiene”. Data was analyzed by descriptive statistics. All analyses were carried out with IBM SPSS Statistics for Windows, Version 21.0 (SPSS IBM, New York, USA).

### Ethical approval

The study was approved by the Regional Ethics Review Board in Gothenburg, Sweden. (Dnr: 157–10). Participants were given both oral and written information in line with the four principal requirements of the Helsinki Declaration: autonomy, non-malfeasance, beneficence, and justice [30]. Informed consent was obtained from the ward manager prior to observation.

## Results

Data on HH in relation to anesthetic care was collected during 94 surgical procedures (both elective and acute surgery) and three observation sessions in the preoperative center between 2012 and 2013, periodically, on weekdays during daytime. A total of 2,393 HHOs were recorded during 6,000 minutes (min). The observational sessions in the preoperative center included in all 210 min. The average length of observational session/induction phases was 23.6 min, SD 9.5, range: 5–44 and 110.8 min/full-length operations, SD 54.5. The average length of surgery was 50 min, SD 41.6, range: 5–210 min.

The mean (m) number of HHOs/full-length operations was m = 10.9/h, SD 6.1, (95% CI 9.1-12.9), range: 2.9- 34.0 with an associated 8.4% adherence to HH guidelines. The corresponding figures for the induction phase were: m = 77.5/h, SD 27.4, (95% CI 69.8- 85.2), range: 21–180 with an associated 3.1% adherence to HH guidelines.

The overall adherence to HH guidelines was 5.3%. Table 2 illustrates the adherence to HH guidelines per observed type of surgery and type of anesthesia.

**Table 2:** **2,393 hand hygiene opportunities per type of surgery and type of anesthesia and the adherence to hand hygiene guidelines (%)**

**Type of surgery**	**Observed hand hygiene opportunities**	**Adherence to HH guidelines (n)%**
*General surgery*	1,256	(59) 4.7
*Orthopedic surgery*	507	(36) 7.1
*Pediatric*	112	(3) 2.7
*Urology*	221	(7) 3.2
*Gynecology*	165	(8) 4.8
*Preoperative center*	132	(13) 9.8
**Type of Anesthesia**	**Observed hand hygiene opportunities**	**(n)%**
*General anesthesia*	1,862	(89) 4.8
*Regional anesthesia*	360	(21) 6.1
*Sedation*	39	(1) 2.6
Missing*	132	

*HH opportunities observed in the preoperative center.

As can be seen in Table 3, HH was more common after a procedure than prior to one, including after clean procedures where HH is not required.

**Table 3:** **The number of opportunities (n) for hand hygiene in relation to different care procedures and the adherence (%) to hand hygiene guidelines before and after the procedure**

**Type of indication and observed hand hygiene**	**Adherence to HH guidelines n (%)**	
**Opportunities before and after (n)**	**Before**	**After**
*Administration of intravenous injection, insertion and manipulation of venous or arterial/central lines (n = 470)*	16 (3.4)	42 (8.9)
*Respiratory care, intubation or laryngeal mask (n = 135)*	3 (2.2)	21 (15.6)
*Regional anesthesia (n = 41)*	1 (2.4)	3 (7.3)
*Urinary tract catheterization (n = 11)*	1 (9.1)	6 (54.4)
Preparation of intravenous injections and handling sterile products (n = 325)*	12 (3.4)	13 (3.7)
*Before and after patient contact (n = 727)*	21 (2.8)	
Total (n = 2,393)	5.3%	

*HH implemented after aseptic task, when not required by guidelines.

The distribution of HHO in relation to professional category is shown in Table 4. In Table 5 the observed opportunities during the induction phase and full-length observations and at the preoperative center are categorized according to the WHO conceptual framework “My five moments of hand hygiene”.

**Table 4:** **The number (n) of opportunities for hand hygiene stratified by profession and overall adherence (%) to hand hygiene guidelines**

**Profession**	**Nursing assistant**	**Nurse anesthetist**	**Anesthesiologist**	Surgeon/instrument nurse*	**Student**	**Total**
**n (%)**	105 (6.3)	1,290 (77.4)	191 (11.5)	23(1.4)	57 (3.4)	1,666
**Overall adherence**	(8.6)	(5.5)	(7.9)	(4.3)	(15.8)	(6.3)
Missing						727^1^

^1^ = Data on professional category is missing for moment 1,3 and 4, e.g. before and after patient contact and surrounding.

*Non-scrubbed surgeons and instrument nurses.

**Table 5:** **Hygiene opportunities (n) and adherence (%) during different observations, categorized by “My five moments for hand hygiene”**

**Opportunities for hand hygiene**		**Full length operations**		**Induction phase**		**Preoperative center**		**Sum**
		**n**	**%**	**n**	**%**	**n**	**%**	
*1*	*Before patient contact*			371	2.2			371
*2*	*Before aseptic task*	482	3.5	445	2.2	82	7.3	1,009
*3*	*After body fluid exposure*	308	15.9	287	5.2	48	14.6	643
*4&5*	*After contact with patient or the patient surroundings*	9	-	359	3.6	2	-	370
	**Total**	**799**	**8.4**	**1,462**	**3.1**	**132**	**9.8**	**2,393**

### Disposable glove use

Glove use was indicated in relation to 249 care procedures. Failure to use gloves when indicated was observed in 107 (43%) cases, occurring mostly in relation to the insertion of venous lines (50.5%) and respiratory care (39.3%).

Disposable gloves were used in relation to 242 care procedures. Out of these, 34.3% were clean or sterile before use, and in 65.7% the gloves had already been used and were contaminated. In 76 cases gloves were used without reason and 68 of these cases the gloves had already been used and contaminated.

## Discussion

The present study is one of few [18,23,24] that in detail assess and quantify the practice of HH, i.e. indications and opportunities for HH during routine anesthetic care. The overall adherence to HH guidelines was found to be very low (5.3%) in combination with a high number of HHOs. This relationship between high workload, risk-prone care procedures and low adherence to HH has previously been established by Pittet et al. [31]. Our study showed that the fluctuation of HHOs during the course of the surgery was substantial. During the induction phase the HHOs occurred in an intensive manner (m = 77.5/h), one HHO tightly followed by the next and this within a relatively short timespan (m = 23.6 min/induction phase), this in contrast to when measured during full-length operations (n = 10.6 HHO/h). In comparison with Biddle and Shah [18] reporting 34–44 HHO/h with peaks up to 54 HHO/h, the present study showed much higher rates of HHOs. Inadequate work processes can partly explain this, as more persons than necessary participated in several care procedures creating avoidable HHOs. Moreover, the frequent interruptions when carrying out aseptic procedures lead to recontamination of the hands or gloves as well as several avoidable HHOs during the course of a care-sequence. These results are similar to those reported by Scheithauer et al. [23]. It is important to remember that the HHOs are reported on an aggregated group level. In reality this would mean that during an induction phase of 24 min, 30 HHOs were created on average. With a team consisting of 1 anesthesiologist, 1 nurse anesthetist and 1 circulating nurse working together, this being the most common scenario, the result would be an average of 10 HHOs per induction phase/person.

HHOs occurred most frequently in relation to aseptic/clean tasks, and the adherence rates observed at the preoperative center (7.3%) and during the induction phase (2.2%) and full-length operations (3.5%) were very low. Implementing HH prior to an aseptic task and the use of aseptic techniques protect the patients from transmission of microorganisms between different body sites and from contaminated surfaces [32] via the hands of the staff. Breaks in the aseptic techniques as seen in the present study can result in contamination of stopcocks, hubs, catheters and intravenous drugs, thereby increasing the risks for bacteremia and mortality [29,33,34].

The adherence to HH was found to differ between surgical specialities. The highest rate was associated with orthopedic surgery (7.1%) and lowest with pediatric care (2.7%). The higher adherence rates during orthopedic surgery might be a reflection of the special safety culture associated with this type of surgery. We observed that in contrast to general and pediatric surgery, orthopedic surgery generated extended infection-preventive measures such as staff wearing special surgical dress, and that the door into the OR was kept locked during surgery.

Differences in HH practice were also found between procedures. Highest adherence was recorded after urinary tract catheterization and lowest before spinal anesthesia and respiratory care. In line with other studies [20], HH in the present study was more common *after* than *before* a care procedure. Whitby et al. [35] describe two types of HH behavior in the healthcare setting, based on the *Theory of Planned Behavior; inherent* and *elective behavior.* Inherent hand washing is a learnt behavior from childhood, with the aim of protecting oneself from “bad” germs. This is carried out as a ritual or after feeling the urge based on physical or emotional reasons. Elective hand washing is implemented in the healthcare setting and covers all other HH actions. This behavior is not triggered by a sense of need for HH, for example after holding the hand on the patient's intact skin. The results from the present study suggest that OR staff predominantly (when at all) implement HH based on inherent behavior rather than in relation to evidence for good HH practice.

Previous studies in the OR have presented adherence rates between 2% and 18% [18-20]. Some of these results are not directly comparable with the present study due to differences in methodology; however, a common denominator is the consistent reporting of low adherence to HH routines in the OR setting. Interestingly, the ward’s own data on HH based on the staff’s self-reported adherence during the study period was 73.2%, and the point prevalence measurement made by the manager of the ward showed a 57.5% overall adherence to HH guidelines. This discrepancy once again raises questions concerning the usefulness of self-reported data [36] and indicator-based strategies that can lead to a perceived pressure to report increased rates [37,38]. As Larson has stated “Falsely high reported rates of hand hygiene will undermine incentives to make real, sustainable change” [37].

Gloves were sometimes used instead of HH and when HH was implemented this often occurred in an inconsistent way, for example when not required. Thus both under-use and over-use of gloves were observed, which might reflect poor understanding of when glove use is indicated, but could also be a result of the socialization process and peer pressure [39]. It has been suggested that using double gloves could reduce the environmental contamination in the anesthetic work area during the induction phase [40]. Since glove use previously and repeatedly has been associated with non-adherence to hand hygiene guidelines [39,41-43] this does not appear to be an optimal method. The risk of transmitting microorganisms via double-gloved hands is just as high as via single or ungloved contaminated hands, i.e. if the gloves are not removed directly after for instance intubation.

It is important to highlight that patients in the OR are in a very vulnerable situation. Due to sedation and medical conditions they are not in a position to protect themselves from harmful events, which means that we have a protective responsibility. However, “blame and shame” have not proved to be effective means to improve safety [44]. We believe that understanding the premises for patient safety and how the work is actually carried out during routine anesthetic procedures, as reported in our study, can provide useful information when planning interventions. Even if there exists an extensive body of knowledge on hand hygiene in different healthcare setting these findings may not be applicable to the OR setting. For instance, Steed et al. [45] reported an average of 5.03 HHOs/bed hour for critical/intermediate care in emergency departments and 1.84 in general emergency departments, results later confirmed in a validation study [46]. Interventions to change HH practice have frequently been based on educational approaches, audits, feedback and the use of reminders that have produced short-term, modest effects [47]. Munoz-Price et al. opine that applying *My five moments for HH* in the OR is impossible and suggest that instead of using HH in relation to a specific indication, anesthesia providers should perform HH every 5 or 10 minutes in order not to interfere with anesthesiologist’s work flow [27]. With this approach there is an unfortunate move away from the scientific basis on which adequate HH practice is grounded [29]. This practice can not be recommended if the main goal is to protect the patient and the medical devices from being inoculated with potentially pathogenic microorganisms. A Cochrane review concluded that the quality of interventions studies intended to enhance HH practice is poor and that an urgent need exists for methodologically robust implementation studies [48]*.* More recently, some interesting studies have been published with well-described methodological approaches, such as cluster randomization and theory-based interventions [13,49-52]. Scheithauer et al. [23] have demonstrated that by standardizing work processes the number of HHOs could be reduced and that in combination with education and feedback, HH adherence increased from 10 to 55%. It is possible that in order to create sustainable changes it is necessary to understand HH practice in relation to the broader context of the OR work which is complex and sometimes stressful with rapidly changing conditions in combination with production pressure. Providing safe administration of anesthesia requires vigilance, instant decision-making and the ability to prioritize and handle multiple tasks [53-55] and the work of a single provider is framed and sometimes conditioned by the system in which he/she works [56]. Further studies are required in order to understand the prerequisites for an optimal use of HH during anesthetic care as well as effective implementation strategies.

We recognize that the present study has important limitations. The change of method can be seen as a limitation, e.g. after observation of HHOs during 43 full-length operations, when we focused instead on the induction phase and also included HH opportunities 1) before patient contact, 2) after patient contact and 3) after contact with patient surroundings in concordance with My five moments for hand hygiene. However, this approach gave us the opportunity to describe in detail the task-intensive induction phase. In order to allow for the single observer to remain concentrated the observational session was shortened. The problem when using a single observer is the human limitations when it comes to observing HHOs occurring simultaneously; thus the observer was instructed to concentrate on the aseptic tasks in conflicting situations. Two observers would produce more reliable data but possibly also increase the Hawthorn effect; however every extra person entering the OR will influence the air quality negatively [57,58], and therefore this was not a valid option.

To minimize the Hawthorne effect the observations were carried out over a longer time period allowing the participants to become used to the observer and the observational situation [59].

The occurrence of HHO and HH practice can vary depending on the time of the day [31,46]. Based on the present study we cannot comment on possible differences over the day, since the present study was carried out during office hours. We used the WHO observation method, as this facilitates comparison between studies. The method is comprehensive and covers more HHOs than other methods that usually measure HH only before and after a procedure [60]. This becomes evident when we change from observing before and after a procedure to including all “My five moments for hand hygiene”. Indeed we found that this method worked very well and gave an accurate picture of the occurrence of opportunities for HH during anesthetic care.

## Conclusions

The present study demonstrates that there is compelling evidence for a low adherence to HH guidelines in the OR setting and thus an urgent need for effective improvement strategies. One of the main problems observed was the lack of aseptic techniques during risk-prone invasive procedures, resulting in several avoidable HHOs. We draw the conclusion that any implementation strategy should include education and practical training on how to implement aseptic techniques and the use of gloves during the induction phase thereby enhancing the possibilities for more appropriate use of HH during anesthetic care.
